# 1,3-Diallyl-2-methyl­benzimidazolium bromide dihydrate

**DOI:** 10.1107/S1600536809034175

**Published:** 2009-09-05

**Authors:** Hamid Ennajih, Rachid Bouhfid, Hafid Zouihri, El Mokhtar Essassi, Seik Weng Ng

**Affiliations:** a Institute of Nanomaterials and Nanotechnology, Avenue de l’Armée Royale, Madinat El Irfane, 10100 Rabat, Morocco; b CNRST Division of UATRS Angle Allal Fassi/FAR, BP 8027, Hay Riad, 10000 Rabat, Morocco; c Laboratoire de Chimie Organique Hétérocyclique, Pôle de compétences Pharmacochimie, Université Mohammed V-Agdal, BP 1014 Avenue Ibn Batout, Rabat, Morocco; d Department of Chemistry, University of Malaya, 50603 Kuala Lumpur, Malaysia

## Abstract

The bonds in the five-membered ring of the title hydrated salt, C_14_H_17_N_2_^+^·Br^−^·2H_2_O, are delocalized. The cation lies on a special position of *m* site symmetry such that the mirror plane passes through the imidazol­yl–methyl bond and is perpendicular to the plane of the cation. The anion lies on another special position of 2 site symmetry. The anion and uncoordinated water mol­ecule are linked into a chain by O—H⋯O hydrogen bonds. One of the water O atoms is disordered over two sites of equal occupancy.

## Related literature

For the crystal structure of 1,3-diallyl­benzimidazolium bromide, see: Holtgrewe *et al.* (2009 ▶). For those of the 1-allyl-3-(cyano­benz­yl)benzimidazolium bromide and its hydrate, see: Xu *et al.* (2008 ▶); Xu & Ye (2008 ▶). 
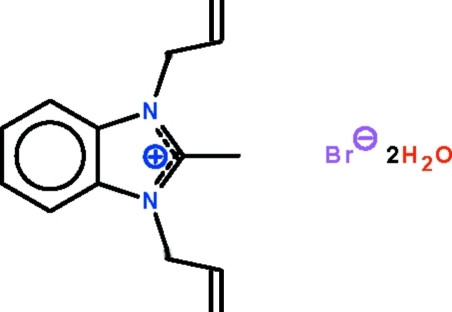


## Experimental

### 

#### Crystal data

C_14_H_17_N_2_^+^·Br^−^·2H_2_O
*M* *_r_* = 329.24
Monoclinic, 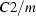

*a* = 13.2888 (2) Å
*b* = 16.8763 (2) Å
*c* = 7.3897 (1) Å
β = 109.773 (1)°
*V* = 1559.54 (4) Å^3^
*Z* = 4
Mo *K*α radiation
μ = 2.64 mm^−1^
*T* = 295 K
0.4 × 0.3 × 0.2 mm

#### Data collection

Bruker APEXII diffractometer
Absorption correction: multi-scan (*SADABS*; Sheldrick, 1996 ▶) *T*_min_ = 0.403, *T*_max_ = 0.590
11870 measured reflections
1851 independent reflections
1497 reflections with *I* > 2σ(*I*)
*R*_int_ = 0.024

#### Refinement

*R*[*F* ^2^ > 2σ(*F* ^2^)] = 0.027
*wR*(*F* ^2^) = 0.085
*S* = 0.95
1851 reflections
103 parameters
6 restraints
H atoms treated by a mixture of independent and constrained refinement
Δρ_max_ = 0.31 e Å^−3^
Δρ_min_ = −0.18 e Å^−3^

### 

Data collection: *APEX2* (Bruker, 2005 ▶); cell refinement: *SAINT* (Bruker, 2005 ▶); data reduction: *SAINT*; program(s) used to solve structure: *SHELXS97* (Sheldrick, 2008 ▶); program(s) used to refine structure: *SHELXL97* (Sheldrick, 2008 ▶); molecular graphics: *X-SEED* (Barbour, 2001 ▶); software used to prepare material for publication: *publCIF* (Westrip, 2009 ▶).

## Supplementary Material

Crystal structure: contains datablocks global, I. DOI: 10.1107/S1600536809034175/sj2636sup1.cif

Structure factors: contains datablocks I. DOI: 10.1107/S1600536809034175/sj2636Isup2.hkl

Additional supplementary materials:  crystallographic information; 3D view; checkCIF report

## Figures and Tables

**Table 1:** Hydrogen-bond geometry (Å, °)

*D*—H⋯*A*	*D*—H	H⋯*A*	*D*⋯*A*	*D*—H⋯*A*
O1—H11⋯Br1	0.86 (1)	2.49 (1)	3.351 (2)	178 (2)
O1—H13⋯O1^i^	0.86 (1)	1.98 (1)	2.822 (5)	166 (4)
O1—H12⋯O1^ii^	0.85 (1)	1.97 (2)	2.748 (4)	152 (3)

Symmetry codes: (i) 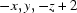; (ii) 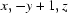.

**Fig. 1.**
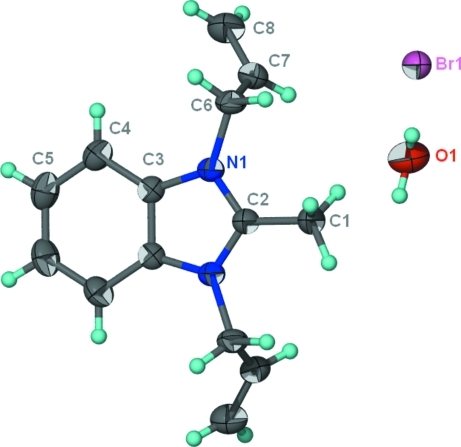
Thermal ellipsoid plot (Barbour, 2001) of [C14H19N2O][Br].H2O at the 50% probability level; hydrogen atoms are drawn as spheres of arbitrary radius. The disorder in the water molecule is not shown. The unlabeled atoms are related to the labeled ones by the x, 1 – y, z symmetry operation.

## supplementary crystallographic information

### Experimental

2-Methylbenzimidazole (1 g, 7.5 mmol), potassium carbonate (1.55 g, 11.25 mmol) and tetra-*n*-butylammonium bromide (0.18 g, 0.75 mmol) were stirred in *N*,*N*-dimethylformamide (50 ml) for an hour. To this suspension was added ally bromide (1.96 ml, 22.5 mmol); the mixture was stirred for two days. The mixture was filtered and the solvent removed under vacuum. The residue was crystallized from ethanol to give yellow crystals in 60% yield; m.p. 516–518 K. The formulation was established by ^1^H– and ^13^C-NMR spectroscopic analysis.

### Refinement

Carbon-bound H-atoms were placed in calculated positions (C—H 0.93 to 0.97 Å) and were included in the refinement in the riding model approximation, with *U*(H) set to 1.2*U*(C). The methyl H-atoms lie on general positions,and are disordered; their occupancies are all 0.5.

The water H-atoms were located in a difference Fourier map and were refined with distance restraints (O–H 0.85±0.01 Å; H···H 1.39±0.01 Å). That hydrogen-bonded to Br1 is ordered whereas the other is disordered over two positions of 0.5 site occupancy. The isotropic temperature factors of the three H-atoms were refined.

### Crystal data

**Table:**

C_14_H_17_N_2_^+^·Br^−^·2H_2_O	*F*(000) = 680
*M**_r_* = 329.24	*D*_x_ = 1.402 Mg m^−^^3^
Monoclinic, *C*2/*m*	Mo *K*α radiation, λ = 0.71073 Å
Hall symbol: -C 2y	Cell parameters from 7035 reflections
*a* = 13.2888 (2) Å	θ = 3.6–24.3°
*b* = 16.8763 (2) Å	µ = 2.64 mm^−^^1^
*c* = 7.3897 (1) Å	*T* = 295 K
β = 109.773 (1)°	Prism, yellow
*V* = 1559.54 (4) Å^3^	0.4 × 0.3 × 0.2 mm
*Z* = 4	

### Data collection

**Table:**

Bruker APEXII diffractometer	1851 independent reflections
Radiation source: fine-focus sealed tube	1497 reflections with *I* > 2σ(*I*)
graphite	*R*_int_ = 0.024
φ and ω scans	θ_max_ = 27.5°, θ_min_ = 2.4°
Absorption correction: multi-scan (*SADABS*; Sheldrick, 1996)	*h* = −14→17
*T*_min_ = 0.403, *T*_max_ = 0.590	*k* = −21→19
11870 measured reflections	*l* = −8→9

### Refinement

**Table:**

Refinement on *F*^2^	Primary atom site location: structure-invariant direct methods
Least-squares matrix: full	Secondary atom site location: difference Fourier map
*R*[*F*^2^ > 2σ(*F*^2^)] = 0.027	Hydrogen site location: inferred from neighbouring sites
*wR*(*F*^2^) = 0.085	H atoms treated by a mixture of independent and constrained refinement
*S* = 0.95	*w* = 1/[σ^2^(*F*_o_^2^) + (0.06*P*)^2^ + 0.349*P*] where *P* = (*F*_o_^2^ + 2*F*_c_^2^)/3
1851 reflections	(Δ/σ)_max_ = 0.001
103 parameters	Δρ_max_ = 0.31 e Å^−^^3^
6 restraints	Δρ_min_ = −0.18 e Å^−^^3^

### Fractional atomic coordinates and isotropic or equivalent isotropic displacement parameters (Å^2^)

**Table:**

	*x*	*y*	*z*	*U*_iso_*/*U*_eq_	Occ. (<1)
Br1	0.0000	0.691870 (15)	0.5000	0.05502 (14)	
O1	0.07379 (17)	0.58143 (11)	0.9005 (3)	0.0787 (5)	
H11	0.055 (2)	0.6089 (13)	0.796 (2)	0.084 (9)*	
H12	0.086 (3)	0.5342 (9)	0.874 (5)	0.075 (18)*	0.50
H13	0.021 (2)	0.582 (2)	0.942 (5)	0.10 (2)*	0.50
N1	0.34476 (11)	0.56471 (8)	0.6215 (2)	0.0381 (3)	
C1	0.1655 (2)	0.5000	0.4821 (5)	0.0557 (7)	
H1A	0.1386	0.4473	0.4841	0.083*	0.50
H1B	0.1472	0.5176	0.3514	0.083*	0.50
H1C	0.1343	0.5351	0.5507	0.083*	0.50
C2	0.2831 (2)	0.5000	0.5747 (4)	0.0393 (5)	
C3	0.45113 (13)	0.54109 (10)	0.7010 (2)	0.0375 (4)	
C4	0.54525 (15)	0.58475 (12)	0.7654 (3)	0.0487 (5)	
H4	0.5454	0.6399	0.7646	0.058*	
C5	0.63843 (16)	0.54125 (14)	0.8307 (3)	0.0562 (5)	
H5	0.7034	0.5679	0.8762	0.067*	
C6	0.30794 (16)	0.64801 (10)	0.5930 (3)	0.0457 (4)	
H6A	0.2385	0.6504	0.4925	0.055*	
H6B	0.3576	0.6791	0.5517	0.055*	
C7	0.30000 (19)	0.68243 (11)	0.7729 (3)	0.0509 (5)	
H7	0.2527	0.6592	0.8254	0.061*	
C8	0.3551 (2)	0.74283 (14)	0.8607 (3)	0.0640 (6)	
H8A	0.4031	0.7673	0.8115	0.077*	
H8B	0.3468	0.7618	0.9729	0.077*	

### Atomic displacement parameters (Å^2^)

**Table:**

	*U*^11^	*U*^22^	*U*^33^	*U*^12^	*U*^13^	*U*^23^
Br1	0.0512 (2)	0.04358 (19)	0.0763 (2)	0.000	0.02951 (16)	0.000
O1	0.0922 (14)	0.0556 (11)	0.0888 (13)	−0.0055 (9)	0.0312 (12)	0.0044 (9)
N1	0.0389 (8)	0.0313 (7)	0.0430 (8)	0.0003 (6)	0.0122 (6)	0.0006 (6)
C1	0.0375 (14)	0.0427 (15)	0.082 (2)	0.000	0.0133 (14)	0.000
C2	0.0392 (13)	0.0351 (13)	0.0433 (13)	0.000	0.0135 (11)	0.000
C3	0.0385 (9)	0.0402 (9)	0.0329 (8)	−0.0015 (7)	0.0109 (7)	−0.0002 (7)
C4	0.0459 (10)	0.0509 (11)	0.0466 (10)	−0.0100 (8)	0.0124 (8)	−0.0048 (8)
C5	0.0398 (10)	0.0764 (13)	0.0470 (10)	−0.0110 (10)	0.0076 (8)	−0.0070 (9)
C6	0.0497 (10)	0.0306 (9)	0.0526 (10)	0.0022 (8)	0.0120 (8)	0.0076 (7)
C7	0.0527 (12)	0.0383 (10)	0.0654 (13)	0.0045 (8)	0.0248 (10)	0.0033 (8)
C8	0.0748 (15)	0.0484 (12)	0.0691 (14)	−0.0005 (11)	0.0248 (12)	−0.0055 (10)

### Geometric parameters (Å, °)

**Table:**

O1—H11	0.86 (1)	C3—C4	1.390 (3)
O1—H12	0.85 (1)	C4—C5	1.379 (3)
O1—H13	0.86 (1)	C4—H4	0.9300
N1—C2	1.339 (2)	C5—C5^i^	1.392 (5)
N1—C3	1.393 (2)	C5—H5	0.9300
N1—C6	1.480 (2)	C6—C7	1.487 (3)
C1—C2	1.479 (4)	C6—H6A	0.9700
C1—H1A	0.9600	C6—H6B	0.9700
C1—H1B	0.9600	C7—C8	1.294 (3)
C1—H1C	0.9600	C7—H7	0.9300
C2—N1^i^	1.339 (2)	C8—H8A	0.9300
C3—C3^i^	1.387 (4)	C8—H8B	0.9300
			
H11—O1—H12	108.7 (16)	C5—C4—C3	115.81 (19)
H11—O1—H13	106.6 (16)	C5—C4—H4	122.1
H12—O1—H13	109.4 (17)	C3—C4—H4	122.1
C2—N1—C3	108.70 (15)	C4—C5—C5^i^	122.17 (12)
C2—N1—C6	126.45 (16)	C4—C5—H5	118.9
C3—N1—C6	124.84 (15)	C5^i^—C5—H5	118.9
C2—C1—H1A	109.5	N1—C6—C7	111.34 (15)
C2—C1—H1B	109.5	N1—C6—H6A	109.4
H1A—C1—H1B	109.5	C7—C6—H6A	109.4
C2—C1—H1C	109.5	N1—C6—H6B	109.4
H1A—C1—H1C	109.5	C7—C6—H6B	109.4
H1B—C1—H1C	109.5	H6A—C6—H6B	108.0
N1—C2—N1^i^	109.3 (2)	C8—C7—C6	123.8 (2)
N1—C2—C1	125.33 (11)	C8—C7—H7	118.1
N1^i^—C2—C1	125.33 (11)	C6—C7—H7	118.1
C3^i^—C3—C4	122.02 (12)	C7—C8—H8A	120.0
C3^i^—C3—N1	106.63 (9)	C7—C8—H8B	120.0
C4—C3—N1	131.31 (17)	H8A—C8—H8B	120.0
			
C3—N1—C2—N1^i^	0.3 (2)	C6—N1—C3—C4	−2.1 (3)
C6—N1—C2—N1^i^	179.79 (12)	C3^i^—C3—C4—C5	−0.5 (2)
C3—N1—C2—C1	−178.4 (2)	N1—C3—C4—C5	−177.78 (17)
C6—N1—C2—C1	1.0 (4)	C3—C4—C5—C5^i^	0.5 (2)
C2—N1—C3—C3^i^	−0.20 (15)	C2—N1—C6—C7	98.5 (2)
C6—N1—C3—C3^i^	−179.67 (13)	C3—N1—C6—C7	−82.1 (2)
C2—N1—C3—C4	177.40 (19)	N1—C6—C7—C8	118.6 (2)

Symmetry codes: (i) *x*, −*y*+1, *z*.

### Hydrogen-bond geometry (Å, °)

**Table:**

*D*—H···*A*	*D*—H	H···*A*	*D*···*A*	*D*—H···*A*
O1—H11···Br1	0.86 (1)	2.49 (1)	3.351 (2)	178 (2)
O1—H13···O1^ii^	0.86 (1)	1.98 (1)	2.822 (5)	166 (4)
O1—H12···O1^i^	0.85 (1)	1.97 (2)	2.748 (4)	152 (3)

Symmetry codes: (ii) −*x*, *y*, −*z*+2; (i) *x*, −*y*+1, *z*.
